# First Danish Single-Institution Experience with Radical Prostatectomy: Biochemical Outcome in 1200 Consecutive Patients

**DOI:** 10.1155/2011/236357

**Published:** 2010-12-22

**Authors:** M. Andreas Røder, Kasper D. Berg, Lisa Gruschy, Klaus Brasso, Peter Iversen

**Affiliations:** Department of Urology, Copenhagen University Hospital Rigshospitalet, Blegdamsvej 9, Afsnit 2112, 2100 Copenhagen Ø, Denmark

## Abstract

Radical retropubic prostatectomy (RRP) as intended curative therapy for patients with clinically localized prostate cancer (PC) was initiated in 1995 in Denmark. This paper reports single-institution results from the first 1200 consecutive patients operated during a 15-year period. Median age at surgery was 63 years. Median PSA was 9 ng/mL. Palpable tumors (≤cT2) were present in 48% of patients. Gleason score at biopsy was ≤7 for 85% of patients. In sixty-five percent of patients, histopathology revealed localized PCa after RRP. Positive surgical margins were found in 39.2% of the cases. Biochemical recurrence (BR) occurred for 214 (18%) of patients. The estimated biochemical recurrence free survival (BRFS) was 71.7% and 63.2% after 5 and 10 years, respectively. When patients were stratified according to the D'Amico criteria, BRFS after 10 years was 75.3%, 59.7%, and 39.3% for low-, medium- and high-risk patients, respectively. In univariate analysis, clinical stage, PSA at diagnosis and type of surgery were significant predictors of BR. In multivariate analysis, Gleason score > 7, PSA > 10, and higher clinical stage were significant predictors of BR. Early Danish results in a population not subjected to screening demonstrate BRFS rates comparable with earlier reports from the prescreening era.

## 1. Introduction

Radical prostatectomy for clinically localised prostate cancer (PCa) was introduced in Denmark in 1995 [1]. Following a slow start, the procedure is now performed in large numbers [2]. Even though early case finding including PSA-based screening has not been recommended, increased public awareness and opportunistic PSA-based screening have gradually resulted in a rising PCa incidence and a shift in stage distribution, with increasing numbers of younger men being diagnosed with clinically localized PCa, suitable for curative therapy [3]. This paper presents results from the first Danish single-institution series focusing on biochemical recurrence-free survival and will compare results with international experiences according to the D'Amico risk classification.

## 2. Material and Methods

Patients with clinically localized prostate cancer (cT1-cT2) and a life expectancy of 10–15 years or more were offered RRP or external beam radiation therapy as curative treatment. RRP was performed according to the method described by Walsh et al. [4]. Robotic surgery (DaVinci) has been performed in a limited number of cases since 2009. 

Patient data have been collected prospectively in a database. Recorded data includes clinical T-category, preoperative PSA, type of surgery, histopathology with Gleason scores, and biochemical outcome. Patients have been staged according to UICCs TNM classification 2002 (patients from 1995–2002 were reclassified). The database is approved by the Danish Data Protection Agency (file no.:2006-41-6256).

Three months neoadjuvant endocrine therapy with LHRH agonists was used routinely in all the first 109 (9.4%) patients. These patients are excluded when analysing final pathology reports. Twenty-two patients were excluded from the analysis of biochemical failure; one patient had cT0 PCa, three patients with preoperatively verified metastatic PC began endocrine treatment already before surgery, and additionally 18 patients had node positive disease when undergoing RRP and initiated endocrine therapy immediately following surgery.

Patients with ≤cT2a, Gleason score ≤6, PSA ≤ 10, and no evident cancer in the apex were eligible for nerve sparing surgical technique. The use of nerve-sparing surgery has expanded recently for selected patients with cT1c, unilateral PCa and Gleason score 3 + 4 = 7 who are now offered contralateral neurovascular preservation. Limited lymphadenectomy in the obturator fossa were performed in patients with PSA > 10 and/or Gleason score ≥ 7 or suspect lymph nodes encountered during surgery. If node positive disease was encountered during surgery, RRP was not performed. 

Postoperatively, all patients were followed with PSA measurements every 3 months for one year, thereafter twice a year for 3 years, and hereafter yearly until BR. 

No patients received neither endocrine nor radiation therapy before verified biochemical failure, defined as the first PSA > 0.2 ng/mL. Time to biochemical failure was calculated from the date of surgery. 

Uni- and multivariate analysis was used to calculate the relative risk of biochemical recurrence according to preoperative patient characteristics. Kaplan-Meier survival analysis was used to calculate the biochemical recurrence free survival and log-rank test to compare risk groups. Analysis was performed with “Medcalc” (Belgium). Results are reported as median and range. *P* values < .05 is considered significant.

## 3. Results

Patient characteristics are shown in Table 1. Median followup was 4 yrs. Median age at surgery was 63 yrs (Range 45–76 yrs). Median age has risen significantly from 63 to 65 yrs through the period (*P* < .0001). Clinically localized PC was present in 1166 (97.3%) patients. One patient with elevated PSA and a massive family history of PC opted for surgery even though repeated biopsies were normal. Final histopathology verified the presence of a Gleason 6 PC in this patient. In 3 of 33 cases with suspected clinically extracapsular extension, metastatic PC had been documented, but RRP was performed RP as part of a debulking procedure. 

Prostate volume assessed by transrectal ultrasound (TRUS) was available in 578 patients (48%) and the median volume was 39 mL (15–150 mL). 

Median preoperative PSA was 9 (0.4–218) (ng/mL) (Table 1). A significant decrease in preoperative PSA (*P* < .001) over time has been found (data not shown). 

Gleason score at biopsy was not available for 123 patients (10.3%) either because they were graded using the WHO system, or the focus was too small for Gleason grading.

Thirty-six procedures (3%) were robotic-assisted RP performed by one surgeon One surgeon performed more than half (635/52.5%) of the open RRPs, while 5 different surgeons carried out the remainder. 

One fourth of all cases (24.4%) underwent nerve-sparing RP. Lymphadenectomy was performed in 682 (57%) of all patients. At final pathology, 771 (64.3%) had pathological confirmation of organ confined PCa, whereas 307 patients (25.5%) were found to have extracapsular tumour extension (Table 2).

Biochemical recurrence occurred for 214 (18%) of evaluated patients. In univariate analysis, PSA > 10 and >cT2 was associated with a significant increased relative risk of biochemical recurrence (BR). Surprisingly, increasing biopsy Gleason scores was not found to be associated with increased risk of BR in univariate analysis. Patients who underwent nervesparing surgical technique had reduced risk of BR. In Cox multiple hazard regression, PSA > 10, Gleason score 8–10 and ≥cT2 were all associated with a significant risk of BR, whereas age and surgical technique had no significant impact (Table 4).

**Figure 1:**
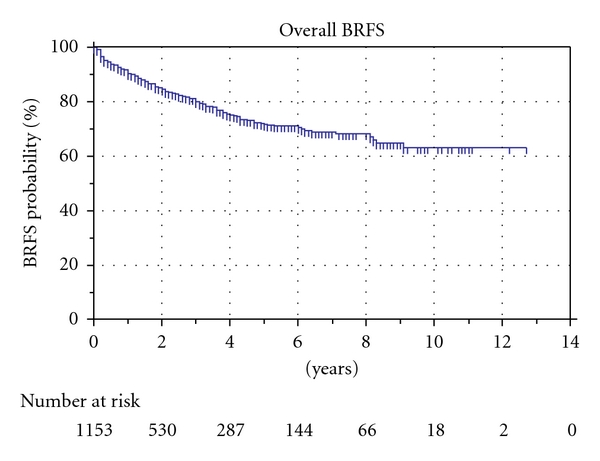
Excluding pT4, pN+, and cT0 patients.

**Figure 2:**
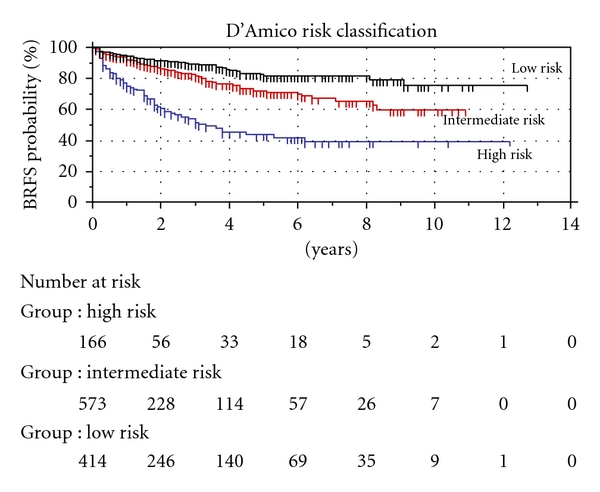
Excluding pT4, pN+ and cT0 patients.

The Kaplan-Meier estimate of biochemical recurrence-free survival (BRFS) was 71.7% and 63.2% after 5 and 10 years, respectively, Figure 1. When patients were stratified according to the to the D'Amico criteria, BRFS after 10 years was 75.3%, 59.7%, and 39.3% for low-, medium- and high-risk patients, respectively, Figure 2. There was a statistically significant difference for the risk of BR between each group.

## 4. Discussion

This paper is the first Danish report on biochemical outcome for patients surgically treated for localized prostate cancer. In Denmark, the approach to curative treatment for localized PCa has been conservative, until early results from the SPCG-4 study reported a significant survival benefit in favour of radical prostatectomy compared to watchful waiting [5]. As a consequence of this approach, PSA-based screening is not recommended in Denmark. This strategy is reflected in the distribution of clinical T-category where almost half of the patients had palpable tumours at diagnosis. This is comparable to other prescreening studies [6–8]. PSA screening will lead to stage migration and modern series often report more than 60% nonpalpable tumours [9].

Median age at RRP has increased, mainly as a consequence of change in treatment strategy as patients >65 years were not offered RRP when the treatment was initiated. 

Median PSA was 9 ng/mL. The median PSA is higher than the comparable reports from the prescreening era. Hull et al. reported a median PSA of 6.8 ng/mL and Boorjian et al. a median PSA of 6.5 ng/mL [10, 11]. In papers where the median PSA is not reported, but the PSA distribution is listed, our patient material also had a lower percentage of patients with PSA < 10 ng/mL [7, 8, 11, 12].

The distribution of biopsy Gleason scores in our series also differs from D'Amico et al.'s paper from 1998 where 77% of patients had Gleason scores ≤6 [12]. The same distribution is comparable to other contemporary American reports [9–11, 13]. We had 48.2% patients with Gleason scores ≤ 6. This is likely to affect the biochemical recurrence rate, especially for the low risk group, and another indicator that our patients had a higher tumour burden than comparable series.

Early reports indicated that neoadjuvant hormonal therapy reduced blood loss, biochemical failure, and positive margin rates after surgery [14, 15]. As later results could not demonstrate any difference on biochemical failure rates [16], neoadjuvant hormonal therapy was abandoned. Neoadjuvant hormonal therapy influences the assessment of final histopathology [17]. Therefore, these patients in our series have been excluded from analysis of pT and pN categories as well as positive margins. 

One fourth of our patients had locally advanced disease on final histopathology. Studies from the prescreening era with comparable preoperative patient characteristics have reported the same rate of extracapsular extension and/or locally advanced PCa [6, 18]. 

As it appears from the cT-category distribution and median preoperative PSA, the patients in our material must be expected to have a higher risk of positive margins than encountered in the reports quoted above. The high rate of positive margins in our series is of concern. A larger tumour burden in this first reported Danish cohort may explain part of this. However, a critical revision of the surgical technique is ongoing, including a meticulous analysis of location and extent of margins and its influence on outcome.

Nerve sparing surgery has been carried out in only 24.8% of patients. Univariate analysis showed a decreased relative risk of BR. This is an indicator of a correct selection of patients.

In our series, lymphadectomy (LND) was performed according to the patients' preoperative characteristics. In spite of our high rate of LNDs we continue to have low and acceptable 30-day morbidity. Moreover, our in-hospital admittance has declined significantly over time from a median of 7 days to currently 3 days [19].

D'Amico risk classification is a model based on pretreatment PSA, biopsy Gleason score, and T-category that predicts the risk of BRFS after definitive prostate cancer treatment. It is important to remember that patients in each risk group have a certain degree of heterogeneity, but the risk stratification has been validated, even in the PSA screening era [9, 10, 20]. 

In the original paper from 1998 [12], D'Amico et al. reported a 5-year BRFS after RRP of 85%, 60%, and 30% for low, intermediate, and high-risk groups, respectively. A later update in 2001 reported the 10-year BRFS of 83%, 46% and 29% for the same groups [21]. Boorjian et al. reported a 10-year BRFS of 82%, 65%, and 55% with patients treated primarily in the PSA screening era [10]. As Hernandez et al. showed in 2007, PSA screening affects the BRFS outcome due to stage migration, lead-time, and lengh-time bias [9]. We report an estimated 10-year BRFS rate of 75.3%, 59.7%, and 39.3%.

The reported differences within each risk group must be regarded with caution. Differences in definition of PSA failure obviously affect the biochemical recurrence rate. We used the first occurrence of a PSA > 0.2 ng/mL as criterion for failure, whereas patients in the D'Amico paper had to have three consecutive rises in PSA > 0.2 ng/mL., likely to postpone the BR occurrence. This was later revised to the second PSA > 0.2 ng/mL [22]. Boorjian et al. used PSA > 0.4 ng/mL as a criterion of failure [10, 12, 21]. Selection of patients also is of great importance. In a screening scenario, the outcome in the low-risk group will be influenced by large numbers of patients with small tumours and possibly insignificant tumours, whereas the distribution of risk factors in the high risk group may be affected by a treatment policy where patients with poor risk factors are offered radiotherapy [9, 10, 21]. 

Multivariate analysis of our patients confirmed the results from the D'Amico paper. We found that patients classified as intermediate or high risk had significantly increased odds of BR.

In contemporary series, positive margin rates have dropped to 10% in centres of excellence [9, 11]. Although the distribution of T-categories, biopsy Gleason scores, and pretreatment PSA indicates a larger tumour volume when compared to contemporary series from countries where radical prostatectomy has been performed for much longer, this may actually be an underestimation of the true difference in biological potential. While the high rate of positive margins in our series is of concern, it is of interest, in continuation of the speculations above, to compare the margin rates from older American series: Boorjian et al. had a positive surgical margin rate of 33%, Bill-Axelson et al. 35.3%, and Blute et al. 34% [7, 10, 18].

## 5. Conclusion

Our series represent the first large cohort of patients undergoing radical prostatectomy for clinically localised PCa in a country where almost no definitive therapy for prostate cancer has been practised before.

## Figures and Tables

**Table 1:** Preoperative patient characteristics.

	Median	Range	%
Age years	63	45–76	
TRUS volume/mL (*N* = 578)	39	15–150	
PSA (*N* = 1198)	9	0.4–218	
PSA ≤ 4			8.9
PSA 4.1–10			51.6
PSA 10. 1–20			30.6
PSA ≥ 20			8.9

cT category		*N*	%
			
T0		1	0.1
T1a/b		35	2.9
T1c		588	49.0
T2a/b		543	45.3
T3a		33	2.7

Gleason score			
≤5		154	12.8
6		432	36.0
7		434	36.2
≥8		57	4.8
N/A^†^		123	10.2

Lymphadenectomy			
Yes		682	57
No		518	43

Surgical method			
Unilateral NS		244	20.4
Bilateral NS		53	4.4
Non nerve sparing		903	75.2

^†^N/A = not available (see text).

**Table 2:** Histopathological data.

		Median	Range
Prostate weight. grams (*n* = 1024)		46	18–236

pT category		*N*	%
pT0		6	0.5
pT2 a/b/c		771	64.3
pT3a		196	16.4
pT3b		110	9.1
pT4		3	0.3
N/A^†^		113	9.4

pN category			
N0		566	47.2
N1		18	1.5
Nx		503	41.9
N/A^†^		113	9.4

pM category			
M0		833	69.4
M1		3	0.3
Mx		251	20.9
N/A^†^		113	9.4

Margins			
Positive		470	39.2
Negative		617	51.4
N/A^†^		113	9.4

Specien Gleason score	≤5	66	5.5
	6	278	23.2
	7	638	53.2
	≥8	72	6.0
	N/A^†^	146	12.2

^†^N/A = not available (see text).

**Table 3:** Uni- and multivariate analysis. Risk of biochemical recurrence.

	RR	95% CI	*P*-value
PSA			
PSA ≤ 4	0.7	0.4–1.3	.25
PSA 4.1–10	1		
PSA 10, 1–20	1.7	1.3–2.3	.0001
PSA ≥ 20	3	2.2–4.1	<.0001

cT-category			
T1a/b	1,6	0.8–3.1	.2
T1c	1		
T2a/b	1.9	1.4–2.4	<.0001
T3a	3.1	1.9–5.0	<.0001
cT0 excluded (*N* = 1)			

Biopsy Gleason score			
≤5	1		
6	0.8	0.6–1.2	.3
7	0.8	0.5–1.1	.2
≥8	1.1	0.7–1.7	.7
N/A = 123			

Age			
<56	0.9	0.6–1.4	.9
56–65	1		
>65	0.9	0.7–1.2	.4

Surgical method			
Unilateral NS	0.5	0.4–0.8	.002
Bilateral NS	0.6	0.3–1.3	.2
Non nerve sparing	1		

^†^ N/A= not available (see text).

**Table 4:** Cox multiple regression hazard analysis.

	OR	95% CI	*P*-value
Age			not significant
Surgical technique			not significant

PSA			
PSA ≤ 4			not significant
PSA 4.1–10	1		
PSA 10, 1–20	1.7	1.2–2.3	.0011
PSA ≥ 20	2.8	1.9–4.2	<.0001

Biopsy Gleason score			
≤5	1		
6			not significant
7			not significant
8–10	3.7	2.4–5.6	<0001
GS N/A excluded			
cT-category			
T1a/b			not significant
T1c	1		
T2a/b	1.6	1.2–2.2	.0014
T3a	2.6	1.5–5.6	.0034

## References

[1] Brasso K, Ingeholm P, Iversen P (2001). Radical prostatectomy: the first consecutive patients operated on at Rigshospitalet. *Ugeskrift for Laeger*.

[2] Brasso K (2007). Prostate cancer—incidence and risk factors. *Ugeskrift for Laeger*.

[3] Brasso K, Ingimarsdottir IJ, Thomassen L, Friis S, Iversen P (2007). Prostate cancer in Denmark 1943–2002. *Ugeskrift for Laeger*.

[4] Walsh PC, Retik AB, Vaughan ED, Wein AJ (2002). *Cambell’s Urology*.

[5] Holmberg L, Bill-Axelson A, Helgesen F (2002). A randomized trial comparing radical prostatectomy with watchful waiting in early prostate cancer. *New England Journal of Medicine*.

[6] Zinoke H, Oesterling JE, Blute ML, Bergstralh EJ, Myers RP, Barrett DM (1994). Long-term (15 years) results after radical prostatectomy for clinically localized (stage T2c or lower) prostate cancer. *Journal of Urology*.

[7] Blute ML, Bergstralh EJ, Iocca A, Scherer B, Zincke H (2001). Use of Gleason score, prostate specific antigen, seminal vesicle and margin status to predict biochemical failure after radical prostatectomy. *Journal of Urology*.

[8] Gerber GS, Thisted RA, Scardino PT (1996). Results of radical prostatectomy in men with clinically localized prostate cancer: multi-institutional pooled analysis. *Journal of the American Medical Association*.

[9] Hernandez DJ, Nielsen ME, Han M, Partin AW (2007). Contemporary evaluation of the D’Amico risk classification of prostate cancer. *Urology*.

[10] Boorjian SA, Karnes RJ, Rangel LJ, Bergstralh EJ, Blute ML (2008). Mayo Clinic validation of the D'amico risk group classification for predicting survival following radical prostatectomy. *Journal of Urology*.

[11] Hull GW, Rabbani F, Abbas F, Wheeler TM, Kattan MW, Scardino PT (2002). Cancer control with radical prostatectomy alone in 1,000 consecutive patients. *Journal of Urology*.

[12] D’Amico AV, Whittington R, Bruce Malkowicz S (1998). Biochemical outcome after radical prostatectomy, external beam radiation therapy, or interstitial radiation therapy for clinically localized prostate cancer. *Journal of the American Medical Association*.

[13] Kattan MW, Eastham JA, Stapleton AMF, Wheeler TM, Scardino PT (1998). A preoperative nomogram for disease recurrence following radical prostatectomy for prostate cancer. *Journal of the National Cancer Institute*.

[14] Schulman CC (1994). Neoadjuvant androgen blockade prior to prostatectomy: a retrospective study and critical review. *Prostate*.

[15] Van Poppel H, De Ridder D, Elgamal AA (1995). Neoadjuvant hormonal therapy before radical prostatectomy decreases the number of positive surgical margins in stage T2 prostate cancer: interim results of a prospective randomized trial. *Journal of Urology*.

[16] Aus G, Abrahamsson PA, Ahlgren G (2002). Three-month neoadjuvant hormonal therapy before radical prostatectomy: a 7-year follow-up of a randomized controlled trial. *BJU International*.

[17] Armas OA, Aprikian AG, Melamed J (1994). Clinical and pathobiological effects of neoadjuvant total androgen ablation therapy on clinically localized prostatic adenocarcinoma. *American Journal of Surgical Pathology*.

[18] Bill-Axelson A, Holmberg L, Ruutu M (2005). Radical prostatectomy versus watchful waiting in early prostate cancer. *New England Journal of Medicine*.

[19] Røder MA, Gruschy L, Brasso K, Iversen P (2009). Early complications following open radical prostatectomy. *Ugeskrift for Laeger*.

[20] Mitchell JA, Cooperberg MR, Elkin EP (2005). Ability of 2 pretreatment risk assessment methods to predict prostate cancer recurrence after radical prostatectomy: data from CaPSURE. *Journal of Urology*.

[21] D’Amico AV, Whittington R, Malkowicz SB (2001). Predicting prostate specific antigen outcome preoperatively in the prostate specific antigen era. *Journal of Urology*.

[22] D’Amico AV, Whittington R, Malkowicz SB (2002). Biochemical outcome after radical prostatectomy or external beam radiation therapy for patients with clinically localized prostate carcinoma in the prostate specific antigen era. *Cancer*.

